# Extra Supplement—The Nursing Mirror

**Published:** 1890-06-21

**Authors:** 


Being the Extra Nursing Supplement of "The Hospital" Newspaper.

All Contributions for this Supplement should be addressed to the Editor, The Hospital, 140, Strand, London, W.C., and should have the word "Nursing" plainly written in left-hand top corner of the envelope.

### En Passant.

**Short Items.**—Lady Alexandra Leveson-Gower has entered the nursing profession, and is now working at St. Bartholomew's Hospital.—At the Free Christian Church, Clarence Road, on Sunday evening, the subject of the sermon was "The Works of Florence Nightingale."—We are glad to hear that the Nurses Hostel is so successful, rooms have to be engaged some time beforehand.—Luton Cottage Hospital authorities have decided not to start a nursing institution because "it was generally deemed to involve financial loss!"—The death is announced of the superior of the Sisters of St. Vincent de Paul, who nurse the Italian Hospital, Queen's Square.—Lady Victoria Lambton contributes an article to last week's *Queen* on Rural District Nursing.—Miss Curno and Miss Greenstreet (Sister Mark of Guy's) sign a circular in favour of the convalescent home the Santa Claus Society wants to start.—Miss Alice Robson read the report of the 1st annual meeting of the Ladies' Nursing Guild of South Shields. Over 500 visits were paid during the year.—*The Lady* must look to its laurels: it used to have very good articles on nursing, but has lately descended to an extraordinary dissertation on marriage under the head of "Pen and Ink Sketches of Hospital Life"!—A hospital in Iowa was destroyed by fire on June 12. Owing to the coolness and prompt energy of the nuns, 40 helpless patients were carried to places of safety. Only one life was lost, that of a nun known as Sister Mary Irene, who perished while endeavouring to save some precious relics from the hospital chapel.

**Excursions.**—Here are a few hints for summer afternoons for those nurses who appreciate fresh air. Take a shilling return ticket to Chingford from Liverpool Street, walk a little way into the Forest, and lie down under the trees and think of nothing. When hunger recalls you from this serious form of meditation you will find plenty of places handy where a cheap tea can be obtained. This excursion is best taken in the middle of the week when the trains are not so crowded. Take a shilling single ticket from Waterloo to Kingston, walk up the towpath beside the Thames to Hampton Court; the *Cardinal Wolsey*, a delightfully-comfortable steamer, leaves Hampton Court about four in the afternoon, and you can either return to town by it, or, if time is short, go as far as Richmond on the steamer, and train from there home again. For those nurses only free on Sundays, we may mention that Hampton Court Palace and Picture Galleries, and Kew Gardens (free) are both open on the Day of Rest. Kew Gardens can be reached by the underground railway, and after wandering about the huge palm-houses a nurse can find a pretty quiet spot by the river's bank to sit and rest till duty calls her home again. The underground railway has lately crept out and away from its City haunts, and penetrated into all sorts of quaint and charming nooks. You can get away down to Rickmansworth and Chesham, and then you can 'bus across to sleepy old Amersham. Perhaps the best way of penetrating into Bucks, though, is to go by the 'bus which daily leaves the Old Bell Inn, Holborn, at three o'clock, and gets to Missenden about sunset. If you secure outside seats on this vehicle, and the day proves fine, you are bound to enjoy yourself. Is it superfluous to mention such Cockney, but charming, resorts as Hampstead Heath and the grounds of the Crystal Palace?

**Our Infirmary Baby.**—An interesting event has just taken place in the chapel of the Royal Infirmary, Preston, the baptism of a little girl, born in the Alice Ward. Her mother was brought in about a fortnight before her birth suffering from a fractured patella. Under the circumstances it is not surprising that baby has excited an unusual amount of interest from all in the institution, and her baptism at evensong, on Whit-Sunday, was made the occasion of a general rejoicing. The chaplain, the Rev. C. O. L. Riley, performed the rite, the appropriate service, the special hymns, even the baptismal robe had all been carefully prepared with great forethought by the matron and nurses. Baby had several of her own relatives present, including her father and Godfather, two of the nurses for her Godmothers, and of course was christened Alice Ward, while—strange coincidence!—her surname is Preston. Afterwards all were entertained by the kind and thoughtful matron, Miss Pigott, at a festal repast spread before the bed of the injured but happy mother, and the crowning feature of the board was a large snow-white christening cake. Baby is thriving fast, and seems indeed blessed in the novel position of "Our Infirmary Baby."

**Matron and House-Surgeon.**—A very serious correspondence is going on in the *Macclesfield Courier* on the subject of the management of the infirmary, complaints having arisen that the matron has too much authority and interferes in the department properly appropriated by the house-surgeon. We are glad to say the matron has taken no part in the controversy, though the late house-surgeon, Dr. A. E. Nuttall, has been very busy with his pen. Mr. Nuttall quotes the two following examples:—"'Rule 105—The patients' friends shall be allowed to visit them on Wednesdays and Saturdays, from three to four o'clock in the afternoon. No person shall be admitted to visit a patient on other days without the permission of the house-surgeon.' When house-surgeon I discovered that the matron was in the habit of giving this permission, and referred her to the rules, telling her that she had not the power to give the patients' friends leave to go into the wards. The matron then brought the matter before the House Committee who upheld her authority on the ground that a notice had been issued by them, in which, amongst other things, the following was found—'No person shall be allowed to visit the patients without the permission of the house-surgeon or matron.' 'Rule 54: The senior house surgeon shall regularly and accurately, on the day of each order, enter in a book provided for that purpose, the various stimulants ordered, and names of the patients for whom they are ordered, and present the same at each meeting of the House Committee.' On three separate occasions I brought the question of the consumption of stimulants before the notice of the Committee, as, for various reasons, I was not at all satisfied about it. In case of any inquiry, the house-surgeon would be responsible, and he had no means of knowing where the stimulants went to, as, in defiance of the above rule, the matron had possession of the book referred to, and did what was required, and the House Committee wished her to have it and do what was necessary, rather than the house-surgeon." It is noteworthy that "a little friction" constantly exists in provincial hospitals between the matron and the house-surgeon, and committees therefore cannot be too exact in drawing up rules and maintaining them.

### Sketches of Insanity.

By FRANCIS H. WALMSLEY, M.D., (Medical Superintendent of Leavesden Asylum).

### I.—INTRODUCTORY.

"Oh! she was changed
As by the sickness of the soul; her mind
Had wandered from its dwelling, and her eyes
They had not their own lustre, but the look
Which is not of the earth; she was become
The Queen of a fantastic realm; her thoughts
Were combinations of disjointed things;
And forms impalpable and unperceived
Of others' sight familiar were to hers.
And this the world calls Frenzy!"
"The Dream."—BYRON.

BEFORE proceeding to the study of insanity we must glance at the phenomena of healthy mind; the importance of so doing will be manifest when we come to deal with the delusions and false beliefs of the insane.

For our present purposes we may say that the mind at our birth is a blank—is like a sheet of white paper upon which experience writes our ideas.

The growth of intelligence is due to the repetition of experiences.

For descriptive purposes the mind may be divided into—

1. Sensation or Perception.
2. Intellect or Thought.
3. The Emotions or the Feelings.
4. Volition or the Will.

Let us examine more closely each of these divisions :—

I.—Sensation : By the different sensations we experience we are made acquainted with the existence of the external world and the objects it contains. Impressions are made upon the senses or gateways of knowledge, as they are aptly called. These impressions are transmitted to the brain, which thus becomes stored with ideas.

The senses are: Smell, taste, hearing, sight, touch, and the muscular sense.

By the muscular sense we obtain a correct view of the situation of different parts of the body; it is the sense also by which we appreciate resistance, weights, &c.

When false impressions of any of the senses are generated in the mind, delusions are developed.

II.—The emotions or the feelings : All that goes to make up pleasure or pain, happiness or misery, and whatever is called mental excitement comes under this head. When our feelings are at all strong they manifest themselves in outward demonstrations and physical effects, hence we have the smile, the frown, the cry, the groan, or tears. The emotions are exemplified in wonder and awe, hope and despair, self-esteem and humility, courage and fear, love and hate; grief and disappointment give rise to anger, anger to envy, envy to malice, and malice to grief again. In like manner our temper, when elevated with joy, naturally throws itself into love, generosity, courage, pride, etc.

III. Will : This involves *action*, or the putting forth of power for some end. All our energies or voluntary activities are exercised in the pursuit of pleasure and in the avoidance of pain; to securing whatever is beneficial and conducive to our welfare, and to avoiding whatever is calculated to injure or destroy. The influence of the will is most powerful over the muscular system of the body, next over the thoughts, and least of all over the emotions.

IV. Intellect or thought: The mental faculties are memory, reason, judgment, imagination, etc.

*Memory.*—The faculty of the mind by which it retains the knowledge of past events. Some people have peculiar ability in remembering names, others places, others dates, others incidents.

*Reason.*—As the individual so regulates his conduct and orders his actions that they tend to the furtherance of life or to its hindrance, is his intelligence ranked as high or low. In the former case he is popularly said to have all his wits about him, in the latter he is said to be bereft of reason.

*Imagination.*—That faculty of the mind which forms new combinations of ideas, from the materials stored up in the memory. Imagination can form monsters and form incongruous shapes and appearances. The exercise of imagination in the study or in the construction of works of art is fitted to have an ennobling or refining effect upon the mind.

By the exercise of the imagination we can conceive it possible that "as the moulding and remoulding of man and society into mutual fitness progresses, and as the pains caused by unfitness decrease," a time may come when mankind will be in complete accord with his environment, and the frenzy of the mind will be no more!

*(To be continued.)*

### The Nurses' Bookshelf.

### A HISTORY OF DISTRICT NURSING.*

THIS book is dedicated to the Queen, whose large donation from the Women's Jubilee Offering to the cause of district nursing has done much to bring forward the merits of this arduous branch of our calling. The introduction is written by Florence Nightingale, and deals with the moral training necessary to a nurse. In a few noble and pointed sentences, Miss Nightingale demonstrates how the nurse may make the desolate and poverty-stricken home to "blossom like the rose;" how she may be the "still small voice" which may do more to crush the demon of poverty than any amount of strikes or legislative panaceas. "We hear much about contagion and infection of disease," writes Miss Nightingale, "may we not also come to make health contagious and infectious?" Certainly our district nurses have the chance of planting many a health germ or maxim in good ground, if only these germs will grow and spread apace. Very strongly is it urged that there is no formula of nursing, that it cannot be numbered or registered like arithmetic or population, or tested by public examination; nursing has to nurse living bodies and spirits, and must be sympathetic; it is work for the heart as well as the brain. The history of district nursing is only a history of thirty years; it was in 1859 that the first district nurse commenced work in Liverpool, and within four years the whole of Liverpool had been divided into eighteen districts, and each supplied with a nurse working under trained superintendence. This success caused the system to spread, and in 1868 the East London Nursing Society was founded; in 1874 the Metropolitan and National Nursing Association was established. At the desire of Miss Florence Lees, the nurses of this last association were all gentlewomen, an experiment which worked thoroughly well. The founding of the Queen Victoria Institute is the last note in the progress of district nursing; indeed, the whole subject is so modern that it seems strange to speak of a "history" of it. Most of us can remember all the chief points gained by this estimable form of charity. The appendices to the book include the report of the Provisional Committee of the Queen Victoria Institute to the Trustees, the first report of the Scottish branch, the conditions of affiliation, the requirements in nurses who work for the Institute, and other useful information. To those desirous of establishing Queen Victoria nurses in their districts, this book will be most useful.

* "Sketch of the History and Progress of District Nursing." By William Rathbone, M.P. (London: Macmillan and Co.)

### A Nurse in a Lodging House.

Who among City people, when wandering along some close, dirty, muddy street, has not had their attention arrested by either lamp or sign-board, intimating the house of shelter for poor strangers, or perhaps better known by the name of model lodging-house? Duty obliging me to spend a week inside one of these homes, and being at liberty to wander over the establishment asking as many questions as I chose, I may be able to give a little information of what goes on in the interior, which may prove interesting to some of your readers less fortunate than myself. The home in question has accommodation for about three hundred and sixty men; it is situated near to the docks, and surrounded by a poverty-stricken locality. Outside the door the street swarmed with groups of men, some of them lounging against the wall smoking and chewing tobacco, while others sat upon the pavement and dozed away in the sunshine. Entering the door a through passage is found, almost lined with the poorest specimens of humanity; to the right of the passage are two small windows; from the one tickets for beds are sold, the price being threepence, fourpence, or sixpence a night. Behind the other window is a shop where groceries, vegetables, bread, milk, &c., are sold, and in quantities from the worth of a farthing upwards. Turning to a door on the left, we enter a large hall, or general sitting-room, there being a smaller room set apart for those paying a sixpence a night, and spoken of by the others as "the gentlemen." This hall is wide, long, and airy, with numerous wooden forms set round. The daily newspapers are brought, pasted on boards, and hung up here, where they are eagerly read and discussed —especial attention being given to petty thefts, strikes, and the latest murder trial. After a due amount of swearing, quarrelling, and probably a few sharp blows, those who are in possession of a few coppers will retire for a snooze on one of the forms, while their fellows in less independent circumstances go in quest of a job. At the farther end of the hall we enter the kitchen, which is furnished with a huge hot-plate; plenty of pots, pans, and tin cans for brewing tea, with two women in charge. Here the inmates bring their eatables to cook, each individual cooking his own. At times the plate is literally covered with all sorts of savoury messes, from stewed steak to a roasted herring, tea and toast being always in abundance. But woe betide the unwary one who has ventured too far from his dainty morsel, for on his return, alas! he finds it has mysteriously disappeared. Adjoining the kitchen is the dining-hall, where they are obliged to eat all their meals, and then return the dirty utensils to the kitchen women, who do the washing-up. Here are a number of small cupboards (barely a foot square) with doors all numbered, each person claiming the cupboard bearing the same number as his bed; therein they can dispose of food, or anything they want kept, till the next meal. We must now return to the passage at the entrance, and get one of the warders to unlock the door leading upstairs. Ascending a narrow staircase, we enter a long gallery with high ceiling and many windows, and containing a lavatory at either end. This whole storey is divided into two galleries, with rows of sleeping apartments on each side. These cubicles are made of wood, standing about eight feet high and open on the top. Inside is a bunk, a high stool, and sufficient standing room to undress comfortably, and the door bolts on the inside. These apartments, I was told, are inhabited by the gentlemen. The second and third storeys are found divided like the first, and contain the fourpence and threepence a-night beds; the only difference being that the ceilings are probably a little lower and the gentlemen have two sheets on their beds, while their poorer neighbours have only one. There are two women employed in each storey to make the beds and clean. All the floors are well scrubbed, and every corner looks clean and airy through the day, but I should think very close at night if all the beds are occupied. I was informed that it is absolutely necessary to have a thorough cleaning every three months; blankets, mattresses, and everything washed and disinfected; and the whole gallery fumigated. Indeed, I can vouch for the necessity, and, by way of friendly advice, would recommend anyone purposing a visit to wear only the shortest of skirts.

Everyone must be upstairs by eleven o'clock, but it would be erroneous to say all went to sleep then, for I have heard sounds, strongly suggestive of being very much awake, at a later hour. One warder is on the alert all night to keep the peace, and be in readiness to open the door. It is not surprising that they should occasionally have nocturnal visitors in search of an Artful Dodger. The morning bells begin ringing at five o'clock, and ring every hour till eight. Everyone must be downstairs by nine, and the door locked for the day to all but the female cleaners, who, in turn, must be finished by eight p.m., so as to admit of the men getting up.

Undoubtedly, the comforts of such a home are great when compared with many a city hobble, and I was informed some men had been in the house for eight and nine years, and were very respectable, though poor; but with the far greater number of inmates alcohol was the curse. Under that same roof we find the lad who passed a bright University career with honour, but is in middle age a wreck; the lad who was nursed in the lap of luxury, but in old age reduced to beggary; the man who has travelled, and admired the beauties of many countries, but now tottering in depravity; the young lad brought there night after night by the man he was taught to call father, but in truth a fiend, and associating with individuals sunk to the deepest in crime and vice. But it is hard for man to judge the poor; I found the inmates of this place civil and respectful when kindly spoken to, yet having a great objection to be preached to.

The Charity Organization Society sells tickets to the charitably-disposed in connexion with this home. On presenting these tickets at the home, they obtain food or lodgings to the full amount, and therefore get the benefit of what might otherwise have gone to the gin-shop. Gregory.

### Examination Questions.

### PRIZE ANSWER FOR MAY.

Diphtheria is infectious through the deposit on the throat and through the breath. It is the duty of the nurse to see that everything not needful to the comfort of the patient, and everything that cannot be disinfected is removed from the room, but at the same time the room should be made to look bright and cheerful. A sheet wrung out of a solution of carbolic acid should be hung over the door; this helps to prevent the spread of infection. The air of the room must be kept at a temperature of from 65 deg. to 70 deg. F., and moist by means of a steam-kettle. The air must not be allowed to become foul but must be kept pure by proper ventilation. The nurse should wear such garments as can be washed and will not be likely to carry infection. The bed should be so arranged that the nurse may be between it and the door and the window, and not on the side next the fire-place; as the air entering by the door or window passes over the bed towards the fire-place laden with diseased germs. Complete isolation is necessary. Especial care must be taken to avoid the patient's breath, particularly when he is coughing. All vessels used to receive the expectoration should be kept thoroughly clean and well disinfected; they should contain disinfectant. Pieces of soft rag (previously disinfected with carbolic acid, Condy's fluid, etc.,) should be used to wipe the discharge from the nose and mouth. They must be immediately burned. All drinking vessels used by the patient should be kept separate. After the recovery of the patient, the room and all the contents must be thoroughly disinfected.

### Everybody's Opinion.

*[Correspondence on all subjects is invited, but we cannot in any way be responsible for the opinions expressed by our correspondents. No communications can be entertained if the name and address of the correspondent is not given, or unless one side of the paper only be written on.]*

### FEVER NURSES.

Nurse Lilly writes: Will you allow me through your paper to say a few words respecting fever nurses? I have taken your Hospital for some years now but have seen very little praise or sympathy in their brave work of love. In my opinion they are a class of women deserving the warmest sympathy, as theirs is a hard and trying life, being so isolated from the rest of the world and their own lives in daily peril. Anyone who has nursed a malignant case of scarlet fever or diphtheria, will know what danger there is to the nurse, and yet who thinks about her?—no one. She does her duty to the patient without the praise of man, but she knows and feels she has One above watching her, and who will say at the end, "Well done, good and faithful servant, enter thou into my rest." They are considered by some people to be a third class of women, but I have found amongst them some very superior women, who have left good homes to come out in the world and nurse their fellow creatures, and who are deeply hurt at the way they are shunned and the lack of sympathy felt for them.

Will no one speak a word for these noble women who are not afraid to risk their own lives for others? I feel sure it would greatly cheer them and help them in their work to feel they were not quite forgotten by the world.

### Appointments.

[It is requested that successful candidates will send a copy of their applications and testimonials, with date of election, to The Editor, The Lodge, Porchester Square, W.]

Arbroath Poorhouse.—Miss Hannah Edwards, of St. Cuthbert's Poorhouse, has been appointed nurse at this infirmary. There were 38 applicants.

Convalescent Home, Melbourne.—Miss MacGeachie has been appointed housekeeper of the Cottage Convalescent Home for Children at Brighton, near Melbourne.

Oldham Infirmary.—Miss Harriet Jackson has been appointed head nurse of the women's and children's ward of the Oldham Infirmary, where she will have forty-two patients under her care. Nurse Jackson was trained at University College, where she has served for ten years. Her sister was at one time head nurse of the men's wing at Oldham. Nurse Jackson holds excellent testimonials.

Royal Albert Hospital.—Miss G. A. Ramsden has been appointed Sister of the Norman and Adams wards of this hospital at Devonport. Miss Ramsden was trained at Edinburgh, and has acted as Sister at Liverpool Northern Hospital, and for five months as night superintendent at the Derbyshire Infirmary.

### Notes and Queries.

### Query.

(20) *Tissuline.*—Can any reader of The Hospital tell where "Tissuline" may be obtained? Enquirer saw it advertised in your paper three years ago, and purchased some (2s. per quire). Tissuline is a prepared white paper of satin softness and smoothness. No other substitute for gutta-percha tissue has proved so satisfactory and economical, and enquirer regrets having forgotten advertiser's address.

### Answers.

*Outfit for India.*—You need a dozen and a half of all underclothing made in zephyr, cambric, or fine linen. Take plenty of corsets, gloves, &c., as all these trifles are costly out there. Pack your gloves in tightly sealed bottles, and line your packing cases with wax paper to be bought at Whiteley's. A nurse's usual uniform can be worn, only the cotton dresses are worn in the hot season, and the merino during the rain. Let the caps be simple and without strings. A riding habit is almost a necessity.—*Late Indian Sister.*

(10) *Free Passage to Australia.*—A nurse can sometimes get a free passage to Australia by acting as nurse to a delicate lady, or to children, during the voyage. Watch advertisements for such a companion, or apply at the offices of the chief lines that run to Australia.

*** Correspondents whose communications do not appear in the current number are requested to refer to the following week's issue.

### The Fourth of July.

The arrangements for the ceremony at Marlborough House, and also for the evening before are now well advanced. One of the great city companies will probably give the use of its magnificent hall and reception-rooms for the entertainment on Thursday evening, July 3rd. Every nurse who has entered the Pension Fund will have good cause for satisfaction, and those who do not yet belong to it will no doubt determine to join it forthwith. Full particulars of the ceremony and entertainments will be given next week.

### Vacancies.

The following vacancies are announced:

### Nurses.

Belfast Nurses' Home.

Brixton Institute.

Birkenhead Borough Hospital; £25.

Birmingham Eye Hospital; £25.

Blackheath Institution.

Burton-on-Trent Institute; £25.

Chelsea Infirmary; £18.

Cheltenham Hospital.

Clapham Institution.

Croydon Nurses' Institute; £22.

Deaconess' House, Chester.

Epsom and Ewell Cottage Hospital; £20.

General Nursing Institute.

Greenwich Union Infirmary; £20.

Highbury Institute.

Huddersfield Nurses' Home.

Ipswich Nurses' Home; £22.

Levick Institution, Paris.

Lock Hospital, Harrow Road.

Nurses' Home, Stockton-on-Tees.

Manchester and Salford Sick Poor Institution.

New Hospital for Women.

North-West London Hospital; £24.

Ophthalmic School, Hanwell; £26.

St. Cuthbert's Poorhouse; £25.

St. Leonard, Shoreditch, Infirmary (night); £20.

Sheffield Nurses' Home.

South-Eastern Fever Hospital; £30.

Swansea Institute; £25.

Swansea Eye Hospital; £25.

Tyrrell Hospital, Ilfracombe; £18.

West Cornwall Infirmary, Penzance; £15.

West Ham Hospital; £24.

Weston-super-Mare Institute.

Wolverhampton Eye Infirmary £12.

Workhouse Infirmary Nursing Association.

### District Nurses.

Bolton; £25.

East London Nursing Society.

Jersey Nurses' Institute; £25.

Middlesboro' Nurses' Home; £25.

### Probationers.

Barrow-in-Furness Hospital; £16.
Bristol Hospital for Children.
Canterbury Union Infirmary.
Chelmsford and West Essex Infirmary.
Gateshead Children's Hospital.
Halifax Infirmary.
King's Lynn Hospital.
West Ham Hospital.

### Matron.

Halifax Fever Hospital; £50.

### Amusements and Relaxation.

### N.B.—New Word Competition Commenced April 5th, 1890, ends June 28th, 1890.

Proper names, abbreviations, foreign words, words of less than four letters, and repetitions are barred; plurals, and past and present participles of verbs, are allowed. Nuttall's *Standard* dictionary only to be used.

The word for dissection for this, the TWELFTH week of the quarter, being

"STRAWBERRY."

| Names. | June 12th. | Totals. | Names. | June 12th. | Totals. |
|---|---|---|---|---|---|
| Adeline | — | 149 | Agamemnon | 24 | 420 |
| Patience | 24 | 417 | G. E. J. C. | — | 34 |
| Lightowlers | — | 335 | S. E. A. | — | 24 |
| Canary | — | 86 | Jenny Wren | 21 | 383 |
| M. E. S. | — | 37 | Multum in parvo | — | 32 |
| Ambition | — | 36 | Ecila | — | 39 |
| M. W. | — | 217 | Weta | — | 259 |
| Esperance | — | 218 | Henri | — | 161 |
| Judy | — | 37 | Holland | 24 | 416 |
| Reldas | 24 | 417 | T. J. | — | 111 |
| Merenda | — | 29 | Nurse Isabel | — | 17 |
| Lucy Locket | — | 40 | Nurse Faith | — | 9 |
| Camellia | — | 30 | E. S. Z. | — | 10 |
| Qu'appelle | 22 | 398 | G. P. | — | 10 |
| Tinie | 24 | 420 | Stumps | — | 33 |
| Nurse Mildred | — | 190 | Bolton | — | 8 |
| Coralie | 22 | 410 | Jesmur | — | 53 |
| Gladys | 23 | 409 | Ran | — | 17 |

### Notice to Correspondents.

*Second Quarterly Word Competition commences July 5th, 1890.*

N.B.—Each paper must be signed by the author with his or her real name and address. A *nom de plume* may be added if the writer does not desire to be referred to by us by his real name. In the case of all prize-winners, however, the real name and address will be published.

Competitors can enter for *all* quarterly competitions, but no competitor may take more than *one first prize* during the year.

